# Source Anonymity in WSNs against Global Adversary Utilizing Low Transmission Rates with Delay Constraints

**DOI:** 10.3390/s16070957

**Published:** 2016-06-27

**Authors:** Anas Bushnag, Abdelshakour Abuzneid, Ausif Mahmood

**Affiliations:** Computer Science and Engineering Department, University of Bridgeport, Bridgeport, CT 06604, USA; abuzneid@bridgeport.edu (A.A.); Mahmood@bridgeport.edu (A.M.)

**Keywords:** wireless sensor networks, anonymity, context privacy, global adversary

## Abstract

Wireless sensor networks (WSN) are deployed for many applications such as tracking and monitoring of endangered species, military applications, etc. which require anonymity of the origin, known as Source Location Privacy (SLP). The aim in SLP is to prevent unauthorized observers from tracing the source of a real event by analyzing the traffic in the network. Previous approaches to SLP such as Fortified Anonymous Communication Protocol (FACP) employ transmission of real or fake packets in every time slot, which is inefficient. To overcome this shortcoming, we developed three different techniques presented in this paper. Dummy Uniform Distribution (DUD), Dummy Adaptive Distribution (DAD) and Controlled Dummy Adaptive Distribution (CAD) were developed to overcome the anonymity problem against a global adversary (which has the capability of analyzing and monitoring the entire network). Most of the current techniques try to prevent the adversary from perceiving the location and time of the real event whereas our proposed techniques confuse the adversary about the existence of the real event by introducing low rate fake messages, which subsequently lead to location and time privacy. Simulation results demonstrate that the proposed techniques provide reasonable delivery ratio, delay, and overhead of a real event's packets while keeping a high level of anonymity. Three different analysis models are conducted to verify the performance of our techniques. A visualization of the simulation data is performed to confirm anonymity. Further, neural network models are developed to ensure that the introduced techniques preserve SLP. Finally, a steganography model based on probability is implemented to prove the anonymity of the techniques.

## 1. Introduction

Wireless Sensor Networks (WSN) consist of homogeneous, small, and low-cost sensor nodes that have limitations in resources and power [1]. Usually, sensors are used to sense information such as temperature, humidity, and light, which notify the sink through other sensor nodes that act as forwarders to pass data to its final destination. WSNs can also be used for monitoring and tracking applications such as monitoring a species nearing extinction in a national park, tracking a soldier in a battlefield or surveillance of the borders of a country [2]. In WSNs, communication between nodes in transmitting and receiving consumes more power than the computation inside the sensor node itself [1]. Since WSNs have limited resources, a sensor node should only compute basic operations [1]. WSNs can be deployed in remote locations that are not reachable by wired networks such as a hostile environment or a vast forest. Thus, security of sensor networks is demanding, and it needs to be addressed very carefully [3,4]. 

Security issues in WSNs are classified into content threats and context threats [5]. Content security focuses on security of the data itself, and it can be achieved by using confidentiality and encryption techniques, whereas context security such as source location privacy (SLP) focuses on concealing the location of the source node [6]. Anonymity of a node means that this node should be untraceable under any kind of statistical analysis applied by an adversary [7]. Sink Location Privacy is another context security concern. It is preventing adversaries from gaining information on the sink node location. The anonymity of the sink node is a completely different problem. The focus of this paper is only on SLP. Adversaries are restricted to two different types, local and global. Mostly, a local adversary has only a partial view of the network, and it can be countered by modifying the existing routing techniques, whereas a global adversary has a full view of the network and can analyze the entire traffic in the network [8]. Moreover, there are two types of attacks which can be employed by adversaries, active and/or passive attack. An active attack occurs when the adversary attempts to alter the network traffic by modifying the header or the content of transmitted packets or even by injecting new packets into the network to apply some attacks such as denial of service. A passive attack analyzes the network traffic without altering it by observing which sensor nodes are sending packets and which nodes are not. A passive attack is more critical and is harder to detect than an active attack, since it cannot be noticed by the system, because it is not applying any kind of modification. 

In WSNs, devices are often attached to the tracked asset, e.g., a Radio Frequency Identification (RFID) tag. An RFID can either employ an active or passive tag. The active tag has a battery and can send signals to sensor nodes, which can be used to simulate the movement of the asset in different locations in the network, whereas a passive tag does not have a battery and cannot send signals. We only consider passive tags in this paper, because they are cheaper and more applicable to the type of problems for WSNs identified earlier [9]. 

The basic idea behind the proposed techniques is as follows. When a real event is detected, it should be reported to the sink. In order to hide the real event, the network should be injected with dummy packets using as low a rate as possible to confuse the adversary. The low rate is needed to minimize the communication overhead of the network. We develop appropriate algorithms that maintain location and time privacy while minimizing dummy-packet transmissions. The algorithms developed are compared on extensive simulations to demonstrate that they maintain SLP. This is an extended research of the original work in [10]. 

There are many techniques used to counter a global adversary such as separate path routing, network location anonymization, network coding, and dummy data sources [11]. Separate Path Routing (SPR) creates multiple paths from source to sink which means each packet of an event uses different route to destination. Network Location Anonymization works on hiding the identity of the source by using pseudonyms. Network Coding divides a packet into smaller pieces. These pieces will follow different routes to the sink. Dummy Data Sources creates fake sources, which create dummy traffic to hide and obfuscate the real traffic inside. This approach will be used in the proposed techniques, because it provides higher anonymity than other approaches.

Three different techniques, Dummy Uniform Distribution (DUD), Dummy Adaptive Distribution (DAD) and Controlled Dummy Adaptive Distribution (CAD), were developed to provide a high level of anonymity with an acceptable delivery ratio, delay and overhead. DUD does not have a mechanism to reduce the delay, which can take a long time to deliver the real event. However, DAD performance in the case of delay is better than DUD, because it increases the transmission rate of nodes that detect a real event or forward packets of a real event. After nodes transmit all the real packets they have, the transmission rate is reduced for those nodes to make the average transmission rate equal to other nodes in the network. This makes the adversary unable to distinguish the difference between real and fake packets. CAD is an improvement of DAD in the case of the delay because it forces the real packet to be transmitted after n-intervals if the node fails to transmit the real packet within n-intervals. This guarantees the arrival of a real event within a specific time.

The rest of this paper is organized as follows. Section 2 describes the related work. Section 3 presents the network, routing, adversary, and anonymity models used. Section 4 describes the proposed techniques. Section 5 discusses the metrics, which affect the introduced techniques. Section 6 demonstrates simulation and results. Section 7 discusses the anonymity analysis of the results. Section 8 presents the conclusion.

## 2. Related Work 

The phantom flooding technique presented in [12] attempts to create a fake source that attracts the adversary away from the real source. It has two phases: random walk and a subsequent flooding. In the first phase, a message will be directed to a fake source within $h_{walk}$ hops from the real sender's node. The purpose of the second phase is to send the message to the sink using baseline flooding which is a kind of smart version of the flooding. Instead of transmitting every received packet, baseline flooding keeps track of all previously transmitted packets for each node. If a node received a packet that was already transmitted by itself, this node drops the packet right away. This reduces the overhead since each node in the network only transmits one copy of the packet. Another technique is an Angle Based Dynamic Routing Scheme [7], which calculates the inclination angle between the source node and the receiver as well as between the source node and final destination. Based on this calculation, a set of candidate neighbor nodes is selected randomly to forward the event. This set of candidate nodes is going to change every time an event packet is sent leading to multiple paths towards the destination. This tries to mislead the adversary away from the real source. These protocols are very effective against local adversaries, which have a partial view of the network, but they will struggle against global adversaries that have a full view of the network, as increased traffic in a neighborhood could be used to guess the source. 

There are many techniques reported in the literature to counter a global adversary, such as FitProbRate in [13]. In this technique, every node sends dummy messages based on the exponential distribution and if a real event is detected, the real message will be sent using the same kind of distribution, because the exponential distribution has only one parameter i.e., λ=1µ. Therefore, if μ value of the dummy distribution is similar to the μ value of the real distribution, there will be confusion for the adversary in recognizing the difference between real and fake traffic. However, the authors did not mention how the delivery ratio and delay will be controlled in their technique to meet the application’s requirements. Furthermore, this protocol will increase the overhead and decrease the lifetime of the network, because it uses an A-D test every time a real event is detected. The A-D test is short for the Anderson-Darling test, which is utilized to determine if a series of data follows a specific probabilistic distribution. This approach is known as the goodness fit test. 

The General Fake Source (GFS) approach in [9] is another technique to counter a global adversary by simulating the real movement of the real event such as a Panda in different places in the network when the Panda has passive RFID. The basic idea is to generate the traffic of the fake sources instead of generating the fake source itself by using a token to decide which node should be the fake source. Then the fake source will send traffic as it senses a real Panda. Finally, the token will be passed in the network simulating the movement of the Panda. The problem occurs when the Panda is changing its behavior frequently; the adversary can easily detect the Panda, because fake traffic is only generated when a real event is taking place. In addition, they assume that the sink node must be in the center of the network, which is not applicable in many practical applications. Some of the applications require having the sink node on one side or at some other fixed position in the network rather than the center, which makes this technique weaker in terms of source anonymity. 

Another technique is the recurrent clustering mechanism in [14], where clustering is used to reduce the power consumption. Each cluster has a cluster head. Cluster heads coordinate the events in the network. For each node in the cluster, once it detects an event, it is transmitted to the sink; otherwise the node transmits a dummy packet after a specific time instead. Packets are transmitted to the cluster head that has the higher remaining energy. The cluster head forwards the packet whether it is real or fake to the sink. This technique does not guarantee the delay of the event and delivery ratio, which means it is not suitable for real-time or time sensitive applications. One more technique, which is presented in [15], is Fortified Anonymous Communication Protocol (FACP), which transmits a real or a dummy packet every interval and could consume a huge amount of energy and increase the overhead of the network. In addition, only one packet message is considered, which makes their technique unable to deal with multimedia data that consists of multiple packets. Generally, in the literature, most of the papers focus the effort on how to mislead the adversary on both the location and the time of the real event, but rarely consider confusing the adversary about the existence of the real event.

The proposed techniques avoid most of the drawbacks of previous solutions. Some solutions in the literature do not successfully control the delivery ratio and delay of the real events. CAD increases the delivery ratio and decreases the delay without sacrificing the anonymity of the origin. This allows the real event to be delivered within a specific time. Another disadvantage in some of the previous solutions that they assume a central location for the sink in the network. This is not practical for many applications where the sink could be located anywhere. Therefore, we developed our solutions while considering flexible and adaptive sink location in the network. FACP used fake messages in each interval, which causes high power consumption. In our techniques, we avoided this by introducing probability-based techniques, which reduces the number of overhead messages compared to FACP. A major contribution in this paper is introducing three different analysis models developed to prove the performance of the proposed techniques. Many of the current related work techniques do not have an approval of their performance and how they react to a global adversary attack. Those analysis models show that the proposed techniques protect the location and time of the real event by misleading the adversary about the existence of the real event. The models are output visualization, neural network, and anonymity equation. 

## 3. System Models

In this section, we present the network model, routing model, adversary model and anonymity model. 

### 3.1. Network Model

A number of sensor nodes are distributed in the area of interest. Distribution of nodes can be either uniform and random or fixed. They have the same resources, power and computational capabilities. A sensor node collects information about the target object, which falls within its sensing area and transmits this information to the sink node which has more resources, power and computational capabilities than the regular sensor node throughout the network. The sink node can be placed in any position in the network such as on one side of the network or in the center of the network. Sensing area for each node can be calculated as follows:

(1) $$R_{area}=\;\pi r^{2}$$

where $R_{area}$ is the range area and *r* is the transmitting range. In case of forwarding packets from one node to another, all nodes which satisfy the following equation

(2) $${\left(x_{n}-\;x_{source}\right)}^{2}+{\left(y_{n}-\;y_{source}\right)}^{2}<\;r^{2}$$

are considered neighbor nodes of the transmitting node, where $x_{source}$ and $y_{source}$ are coordinates of the source node and $x_{n}$ and $y_{n}$ are coordinates of the receiver node. Sensor nodes can locate their position by using one of the localization techniques [1] in the deployment stage of the network. There are many localization techniques in the literature that used by WSNs to provide each node in the network about its current location and neighboring nodes’ locations. Many of the localization techniques provided in this literature can work just fine as an additive module to our framework. Each application has its own specific requirements such as the lifetime of the network or the maximum delay. The lifetime of the WSN can be divided into time intervals and each interval can have many subintervals. This could be adjusted to meet the application’s requirements. All real and dummy packets are assumed to be encrypted using a shared key between the sender and receiver nodes and, therefore, the message payload is secure. Real and dummy packets are identical in terms of size and structure to avoid size correlation attack by the adversary. When a receiver node gets a packet, it can differentiate between real and fake packets by decrypting using the shared key.

### 3.2. Routing Model

The routing protocol in our proposed model is based on the location of sensor nodes. Therefore, each node should know its coordinates and its neighbor nodes’ coordinates as well. The routing protocol is selecting the next node on the path towards the sink based on the following equation:

(3) $$d=\sqrt[{2}]{{\left(x_{c}-\;x_{sink}\right)}^{2}+{\left(y_{c}-\;y_{sink}\right)}^{2}}$$

where $x_{sink}$ and $y_{sink}$ are the coordinates of the sink, $x_{c}$ and $y_{c}$ are the coordinates of the candidate node, and d is the destination between sink and candidate node. The node with the smallest d value will be the next hop on the path. The location based routing protocol is flexible, and it means if the selected candidate node is out of battery or has a physical damage, the routing protocol will select the second best candidate node that satisfies the minimum d. The whole process will be repeated until the packet is received by the sink. The developed routing protocol does not cause high power consumption. It is implemented to reduce the number of packets, which leads to less overhead. Flooding based routing is not used in this work, because it surges the number of overhead packets and causes high power consumption.

### 3.3. Adversary Model

In this paper only the global adversary is considered, and the model used is very similar to the one listed in [13,16], which is based on a passive, external, and global adversary. Passive adversary means the observer can analyze and collect packets. An external adversary cannot compromise a sensor node physically, whereas a global adversary has a full view of the network as well as unlimited resources and power. The adversary has its own nodes that are deployed to monitor the whole network and generate a high level of statistical analysis. We have further extended the capabilities of the adversary such that it is able to create a data set of many observed intervals for each sensor node during the lifetime of the network. We assume that an adversary can use this data set to analyze the SLP, e.g., a neural network can be trained on this data set and then the network used to expose the existence of the real event, which could lead to the identity of the source node. We also assume the adversary has the ability to visualize the data set by converting it into a binary image and subtract any suspicious pattern, which could point to an existence of the asset. The target of the adversary can be (1) presence of the real event. If the real event exists, then the target becomes (2) the location of the event and (3) the time of the event.

### 3.4. Anonymity Model

Anonymity of the proposed system can be broken into three main parts: (1) Existence of the real event; (2) location of the real event; (3) appearance time of the real event. In order to satisfy all three parts, the first part needs to be tested. Accordingly, the other two parts can be subsequently achieved. For the first part, anonymity of the system can be calculated by the following equation [17]:

(4) $$d\left(\alpha ,\beta \right)=\;\alpha \log_{2}\frac{\alpha }{1-\;\beta }+\left(1-\alpha \right)\alpha \log_{2}\frac{1-\alpha }{\;\beta }$$

where α is the probability of an adversary faultily detecting an asset, which does not exist. β is the probability of an adversary to not detect the existence of an asset. Therefore, in order to achieve anonymity of the first part, the system should satisfy:

(5) d(α,β) ≤ ε

where d(α,β) is the anonymity provided by a specific technique and ε is the anonymity required by the application. The smaller ε is, the higher the probability an adversary will fail to detect the real event.

## 4. Proposed Techniques

In this paper, three different schemes are developed to overcome the statistical analysis of a global adversary. These schemes are Dummy Uniform Distribution, Dummy Adaptive Distribution and Controlled Dummy Adaptive Distribution. All proposed techniques are based on injecting the network with dummy traffic to confuse the adversary. Therefore, reducing the number of dummy packets while keeping a high level of location anonymity of the source sensor node is essential. The notion of all techniques is to divide the lifetime of the network into equal fixed intervals. Therefore, if an asset is detected and a series of real packets needs to be transmitted, then instead of transmitting a real packet immediately after the event occurs, the packet will be transmitted at the end of the interval. This is necessary to avoid the time correlation attack. All other nodes send a dummy packet at the end of the interval based on a probability if they do not have any real packets. When a sensor node receives a dummy packet, the node will discard it right away, but if it is a real packet, the node will add it to its buffer and try to transmit it in the following interval based on a probability. If the real packet is not transmitted in the current interval, it waits one more interval and the node will attempt to send it again based on the probability. This will mean each node in the network has its own pattern for sending real packets, which look completely random to the observer. Since real and dummy packets are sent only at the end of the interval, they should be both indistinguishable for the adversary.

### 4.1. Dummy Uniform Distribution

Injecting a dummy packet in every interval for each node during the lifetime of the network is going to consume a large amount of power and resources. Therefore, the motivation behind DUD is to have the same transmitting constant rate for both real and fake traffic. It works as follows: Each node will throw a random number ($num_{random}$) between 0 and 1. If the number is smaller than the constant rate i.e., $num_{random}<rate_{constant}$, send the real packet, but if there is no real packet in the node's buffer, send a dummy packet instead. By applying this technique, the adversary cannot recognize if the transmitted packet is real or a dummy. Real packets are only created if a real event is detected, and then the node that detected the real event uses the selected transmission rate, for example 0.1, to try to send the real packet every interval. For instance, if a real event was detected between interval 5 and 6, the node will throw a random number between 0 and 1. If this number was less than the selected threshold, which is in our case 0.1, the real packet will be transmitted in interval 6. However, if the thrown number was larger than the threshold, the real packet will not be transmitted, and the node will try to send it in the following interval (interval 7). This process is repeated until all real packets are sent. Dummy packets are only generated if the node does not have a real event. In addition, dummy packets are sent based on a probability. If the thrown number was less than the threshold, a dummy packet is generated and transmitted by the node. Otherwise, the node will not transmit any packets. However, this scheme does not always guarantee the arrival of events’ packets, because the real packets can take a long time to be delivered, or if there is a specific maximum delay requirement for the application. It is obvious that increasing the transmission rate reduces the delay and increases the overhead and, vice versa.

### 4.2. Dummy Adaptive Distribution

In order to increase the delivery ratio of delivered real packets to total real packets, DAD is introduced and it works as follows. All nodes in the network are categorized into dummy nodes and real nodes. In the beginning, all nodes will be considered dummy nodes using a transmitting constant rate as presented in DUD. Dummy nodes do not generate dummy packets in every interval. They only generate and transmit dummy packets if the thrown random number between 0 and 1 is less than the selected threshold (transmission rate). However, if a node detects an event or needs to forward packets of real events, it becomes a real node. A real node will increase the rate of its real traffic by a specific value (Equation (6)). Moreover, it decreases the rate of its fake traffic by the same specific value (Equation (7)) after all real packets are transmitted starting from the interval at which an event occurs until the end of the network's lifetime. The real and fake rates will be increased or decreased based on the selected transmission rate. Real and fake rates of the real nodes are given by the following equations:

(6) $$R_{real}=R_{const}+\;\frac{N_{real}}{I_{total}}$$

(7) $$R_{fake}=R_{const}-\;\frac{N_{real}}{I_{total}}$$

(8) $$R_{const}=\frac{R_{real}+R_{fake}}{2}$$

where $R_{real}$ and $R_{fake}$ are the real and fake rates respectively. $R_{const}$ is the constant rate of the network, $N_{real}$ is the number of real packets and $I_{total}$ is the number of total intervals.

**Figure 1:**
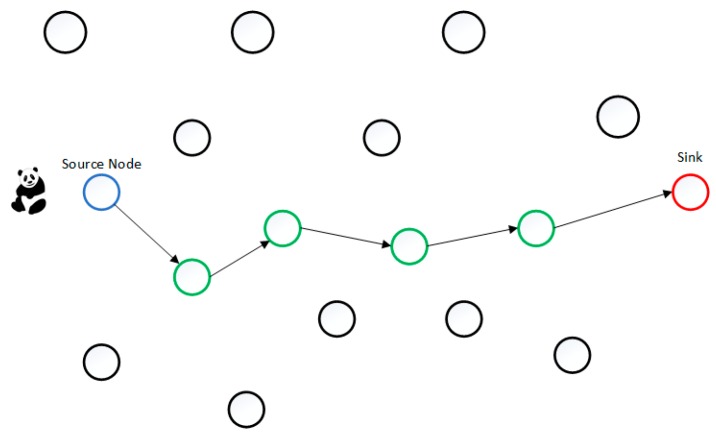
An example of the DAD Technique.

Since the average of real traffic and fake traffic is equal to the original constant rate, the adversary will not notice any change in the rate of the network. Also, *DAD* needs to satisfy the equation

(9) $$N_{real}\leq \;R_{fake}*\;I_{total}$$

in order to perform well; otherwise the average number of the dummy packets will be less than the real packets, meaning that the adversary easily detects the real event. DAD keeps the same level of anonymity as DUD but it will increase the delivery ratio and reduce the delay. However, DAD is still unable to guarantee the arrival of real packets. To clarify DAD, an example is shown in Figure 1. The rate of green nodes will only be modified, because they have real event to report, i.e., forward a packet onward to the sink node. The blue nodes will keep their transmission rate without any change, because they only generate dummy packets.

### 4.3. Controlled Dummy Adaptive Distribution

Since DUD and DAD schemes could fail in delivering real event packets within a certain delay or network lifetime constraint, CAD is introduced to maximize the delivery ratio and minimize the delay to guarantee the arrival of all packets in the real event to the sink within the required constraints. CAD is based on DAD and it increases the real traffic rate and decreases the fake traffic rate using the same Equations (6)–(8) as above. However, if a real node fails to transmit a real packet using the real traffic rate for n-intervals, this node will transmit the first real packet in its buffer without using any kind of probability (transmitting rate is one). Then, it will reuse the original real traffic rate as before for the following real packet. By repeating this process, all real packets can be delivered within guaranteed n-intervals as presented in Algorithm 1.

**Algorithm 1** CAD*constantRate*: select the desired transmission rate*realRate*: from Equation (6)*fakeRate*: from Equation (7)*realIntervalCount*: the maximum number of intervals a real packet can wait*maxIntervalCoun*t: the maximum number of intervals desired by the application*randomSending*: a random number between 0 and 1**for**
$slot_{i}$ < number of lifetime slots **do** **for** each node in the network **do** **if** node has a real packet in its buffer **then** **if**
$slot_{i}$ == sending interval **then** **if**
*maxIntervalCoun*t == *realIintervalCount*
**then** make realRate to 1 **else** make the rate equal to realRate **end if** **if** randomSending ≤ realRate **then** remove the packet from node’s buffer adjust sending time for other packets in buffer add partition to time slot run the desired routing protocol **else** delay all packets in buffer and try next interval **end if** **else** **if** randomSending ≤ fakeRate then send a fake packet **end if** **end if** **end if** **end for** **end for**

## 5. Metrics of Proposed Techniques

There are four different metrics which might affect the performance of the proposed techniques. They are transmitting rate, number of real packets, number of total intervals and delay between real intervals in case of using CAD technique. Using a higher transmitting rate will increase the probability of delivering all real packets within the required delay of the application. In addition, it increases the anonymity of the entire network and decreases the probability of an adversary detecting the real event. However, the higher rate will cause more fake traffic leading to unnecessary overhead. The real event consists of several real packets; the more real packets there are, the more difficult they are to hide inside the fake traffic. Therefore, more fake traffic rate will be demanded, leading to network overhead. However, this metric cannot be controlled because it depends more on the behavior and type of the real event.

The total number of intervals of a network’s lifetime or the maximum delay required by an application plays a very important role in case of delivery ratio of all real packets. The total number of intervals is calculated using the following equation:

(10) $$I_{total}=\frac{t_{s}}{I_{r}}$$

where $t_{s}$ is the simulation time and $I_{r}$ is the interval rate. More intervals increase the probability of real packets’ arrival at the sink, which will improve the overall performance of the network. However, more intervals require more fake packets that will consume resources and energy. The n-intervals is how many intervals the real node will wait before it transmits the following real packet in its buffer without using probability. More intervals mean more security and delay, whereas less intervals means less security and delay. The interval that has a real packet to send is named a real interval. This metric is only valid in the CAD technique.

Selecting the suitable values of these metrics will rely on the requirements of the application. For example, if the application is tolerant in term of delay, we can select as a low rate as possible that satisfies Equation (9). Assume that the real event consists of three packets, simulation time is 100 s, and interval rate is 0.5. The number of intervals is 200 by using Equation (10). Based on the previous numbers. The selected constant transmission rate can be as small as 0.03. This is because the fake rate is 0.015 by using Equation (7). Then, the average of fake packets becomes three after using Equation (9), which is not less than the number of the real packets. Using a smaller transmission rate for instance, 0.02 is not valid in this situation, because it makes the average number of the fake packets less than the number of real packets, which makes it very easy for the adversary to detect the real event. Eventually, it is considered a tradeoff between delay and overhead at one side and privacy at the other side.

## 6. Simulation and Results

**Figure 2:**
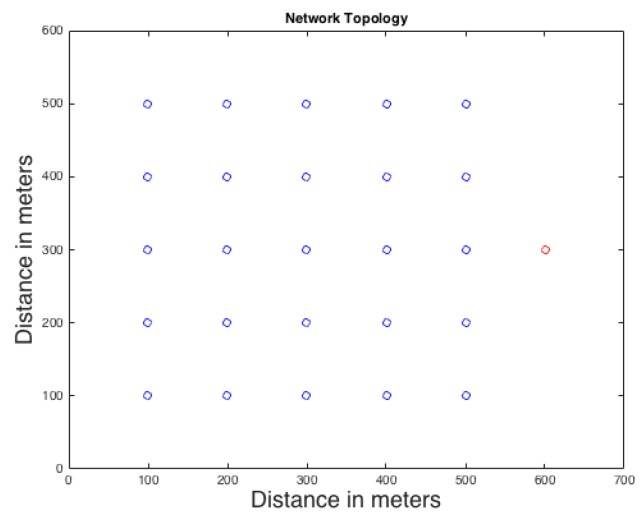
Network topology: blue nodes are regular sensors and the red node is the sink.

In our simulation, the WSN covers an area of interest, 600 m by 600 m. The network consists of 25 sensor nodes and the lifetime of the simulation is 100 intervals. They monitor the movement of an asset such as a Panda. The network has only one sink on one side of the network as shown in Figure 2. If a node detects a Panda, it starts communicating with its neighbors to inform the sink about the current location of the Panda. All sensor nodes have a transmission range of 200 m. The network is injected by different transmitting rates of (0.05, 0.1, 0.15, 0.2, 0.25, 0.3, 0.35, 0.4) That means, if the random number between 0 and 1 is less than the selected transmitting rate, and a real event is received, a real packet will be sent otherwise, a dummy packet will be sent instead. If the random number exceeds the transmission rate, no packet will be sent. For example, if transmission rate is 0.05, then $rate_{realtraffic}$ and $rate_{faketraffic}$ will be 0.08 and 0.02 respectively for DAD and CAD techniques using Equations (6) and (7). Finally, the maximum number of intervals to wait before mandatory transmission in CAD is selected to be 5 (i.e., the maximum wait before the node is forced to transmit the real packet).

**Figure 3:**
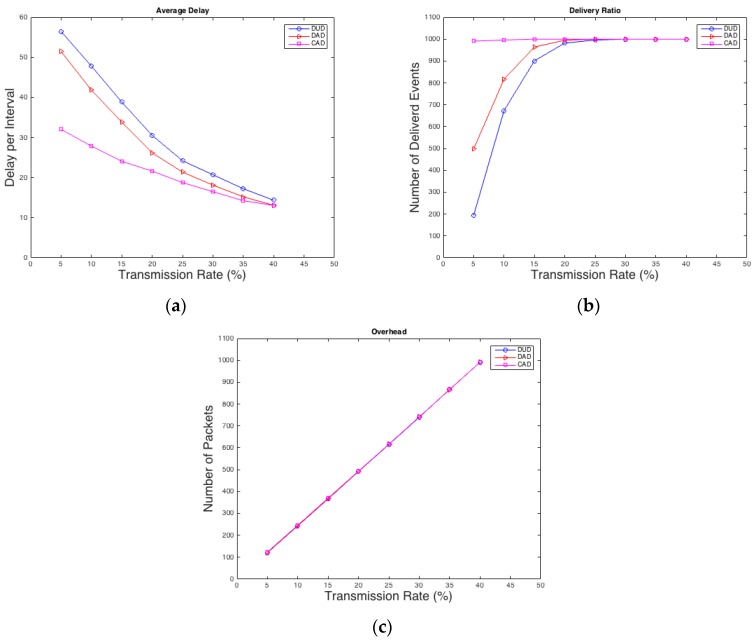
(**a**) Average delay; (**b**) delivery ratio; (**c**) overhead.

Panda can be detected by only one node in the network. One thousand random cases are created for each transmitting rate to evaluate the performance of the WSN. Each case has a random position for the Panda and random starting interval of the event between 0 and 49. A comparison between different rates and how they affect average delay, overhead, and delivery ratio of real events is conducted as shown in Figure 3. All the proposed techniques were developed using C#.

As shown in Figure 3a CAD performs much better in terms of average delay than DAD especially in low rates, which is desired by most of the applications. In case of average delay, DUD is the worst as expected, because it does not have any mechanism to improve the delay of real packets as the other two techniques. Also in terms of delivery ratio, as shown in Figure 3b, CAD is the best and it has an advantage over the other two techniques because it forces the packet of a real event to be sent after a specific waiting time, which is 5 intervals in this experiment. Moreover, DAD is still performing better than DUD since real packets have higher probability to be sent than the dummy ones. Meanwhile, the overhead traffic in Figure 3c is almost the same for all three techniques, because the total number of packets stays the same for all techniques. Thus, it is concluded that CAD and DAD reduce the average delay and increase the delivery ratio without gaining additional overhead.

**Figure 4:**
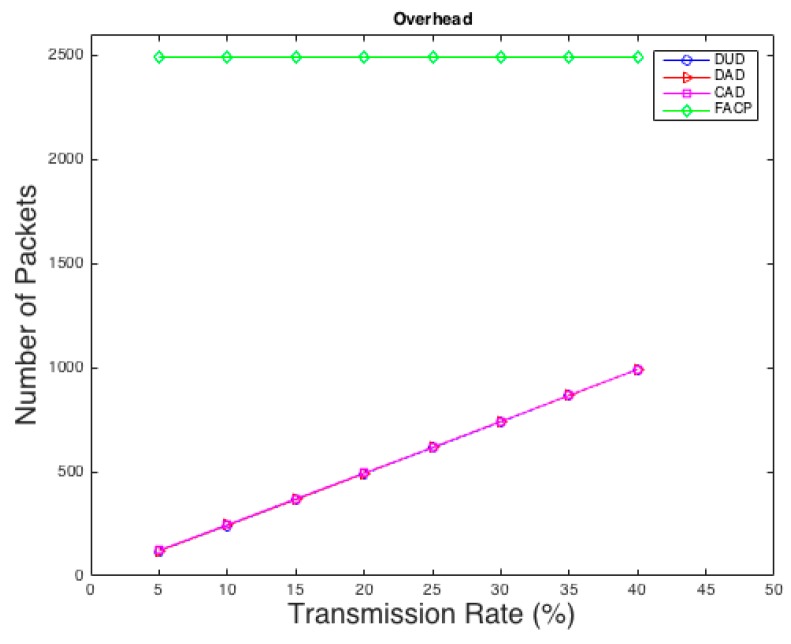
A comparison between the proposed techniques and FACP in case of overhead.

Another comparison is conducted between our techniques and the technique used in [15] in terms of overhead. As shown in Figure 4, our techniques reduce the overhead rapidly compared to the FACP, technique especially in low rates. This is because DUD, DAD, and CAD are probability-based techniques. They create fake traffic based on probability instead of sending dummy packets every single interval like FACP which causes unnecessary overhead. Most of the anonymity applications in WSNs will require low fake rates to reduce overhead to maximize the lifetime of the network.

Results show that CAD technique is able to control the delay and improve the delivery ratio since it uses a forcing mechanism to transmit the real event’s packet after a specific number of intervals. The forcing mechanism is only applied if the transmitting node fails to transmit the real packet using a probability. Moreover, the proposed techniques perform much better than FACP technique in case of overhead. This reduces the power consumption and increases the lifetime of the network. The location of the sink in the experiment is on one side of the network. It can also be placed in any location, which is not valid in many of the previous techniques (they need the sink to be in a particular location in order to perform well).

## 7. Anonymity Analysis

There are many ways to prove that the proposed techniques are increasing the delivery ratio and reducing the average delay without sacrificing the anonymity of the source node. In this paper three different approaches are used to verify this, first by visualizing the output data of the simulations, then by feeding the output data of the simulation to a trained neural network, and finally by applying Equation (4) for anonymity testing as mentioned in the anonymity model described in Section 3. Output data can be converted to a binary matrix. When a sensor node transmits a real or a dummy packet, it is represented by a binary value of 1 whereas, if the node does not transmit, it is represented by a binary value of 0. Since, some of intervals in the simulation will have values of 1 and others will have values of 0, the output of the simulation can be represented as a binary matrix for further analyses. Analysis models were developed and built using MATLAB.

### 7.1. Output Visualization

**Figure 5:**
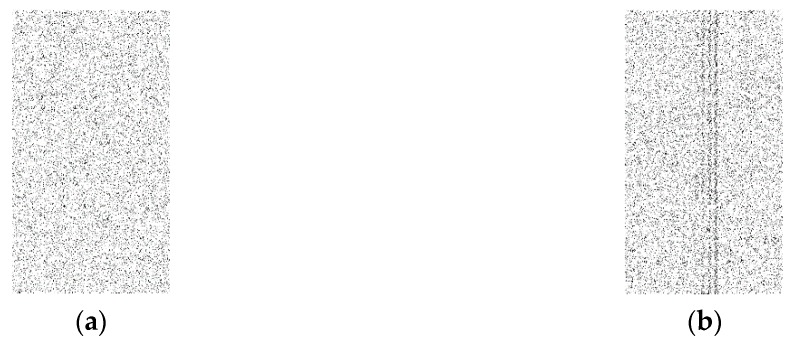
(**a**) is the completely random cases; (**b**) is from a weak technique.

In this approach, the output data, which is represented as a binary matrix, is converted to a binary image. In order to convert the binary matrix into a binary image, ones are represented as black pixels and zeroes as white pixels to make the image visualization better at low rates. The goal here is to check if there is any kind of visible pattern inside the image such as a set of black pixels that look like a row or a column as in Figure 5b, which indicates a real event exists. This is called a weak technique, which refers to any technique that provides a binary matrix that is not random and can be very easily distinguishable from a complete random binary matrix Figure 5a. However, if there is no visible pattern and the image looks completely random, the adversary will not be able to differentiate if a real event exists. In our testing, one thousand random simulation scenarios are created and converted into one image and compared to the images generated by proposed approaches to check if there is any kind of pattern. Different rates of 0.05, 0.1, and 0.15 are evaluated to examine the performance of the proposed techniques.

**Figure 6:**
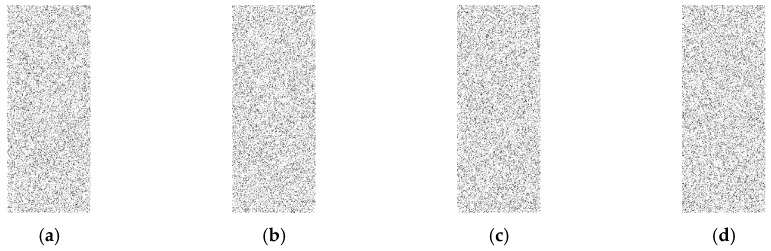
Rate is 0.15, (**a**) is the completely random cases; (**b**) is DUD scenarios; (**c**) is DAD scenarios and (**d**) is CAD scenarios, and all images look very similar to each other.

**Figure 7:**
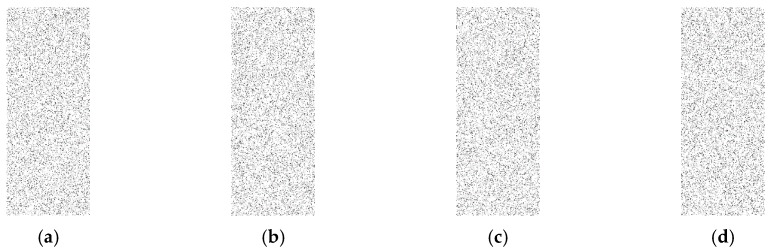
Rate is 0.1, (**a**) is the completely random scenarios; (**b**) is DUD scenarios; (**c**) is DAD scenarios and (**d**) is CAD scenarios, and all images look very similar to each other.

**Figure 8:**
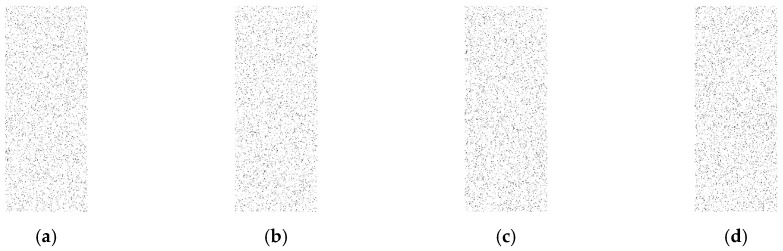
Rate is 0.05, (**a**) is the completely random scenarios; (**b**) is DUD scenarios; (**c**) is DAD scenarios and (**d**) is CAD cases, and all images look very similar to each other.

As shown in Figure 6, Figure 7 and Figure 8, for all different rates used, the proposed techniques look completely random and are very similar to the image with random cases. The figures depicting transmission patterns for the DUD, DAD, and CAD algorithms do not indicate any kind of visual pattern in their images leading to confirm that they have a high level of anonymity.

### 7.2. Output to Neural Network

**Figure 9:**
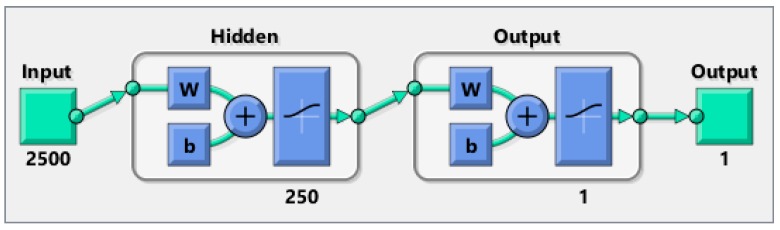
Neural network architecture used in verifying anonymity.

Another way to prove that the proposed solutions provide a high level of anonymity is to create a neural network and train it on many of the patterns (1, 0 matrix) produced by the different techniques. Then, feed an unknown simulation data to the trained network to see if it can detect the occurrence of an event. We compared each approach with the random matrix to see if the neural network is able to recognize the difference or not. If the network can distinguish the difference, this would mean that our solutions have a security flaw. Otherwise, they provide a high level of anonymity. We used two thousand random scenarios as the training input of the neural network. One thousand cases are random scenarios and the other one thousand cases represent one of our solutions (three techniques). The WSN used has 25 nodes and 100 intervals, so 2500 inputs are fed to the neural network. *W* is the weights that are generated by the neural network. At the beginning, those weights are selected randomly, then their values are changed using an algorithm called gradient descent. The aim of the neural network is to find the best weights combination that minimizes the errors between the neural network output and expected output, whereas *b* is just a constant. A graphical representation of the neural network is shown in Figure 9. 

Each technique is tested under different rates which means three rates of 0.05, 0.1, and 0.15 are used per technique. The hidden layer in the feed forward neural network has 250 neurons. A gradient descent back-propagation algorithm is used for training the network. The activation function used is sigmoid for both the hidden and output layer neurons. The output layer has one neuron and produces 0 if there is no real event is detected (Panda) and 1 otherwise. All training data for all experiments achieved almost zero training error. The training and testing results are depicted in the following figures.

**Figure 10:**
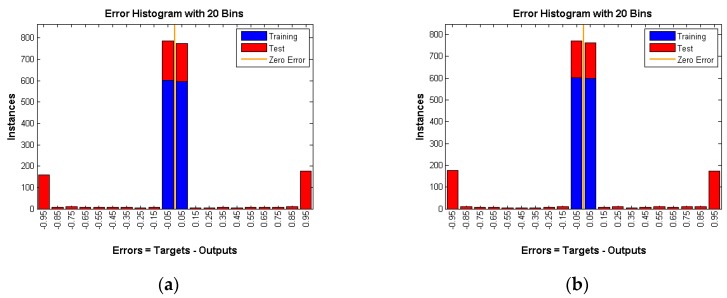

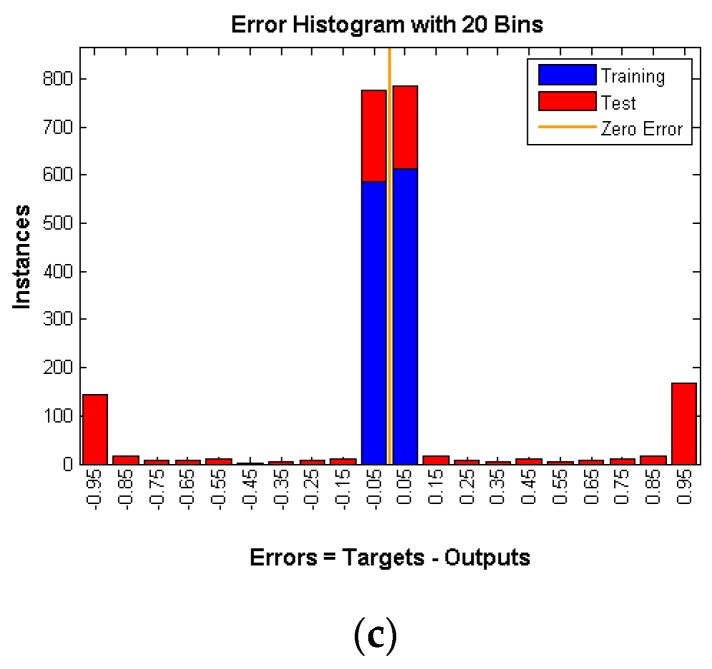
DUD with rate of (**a**) 0.05; (**b**) 0.1 and (**c**) 0.15.

**Figure 11:**
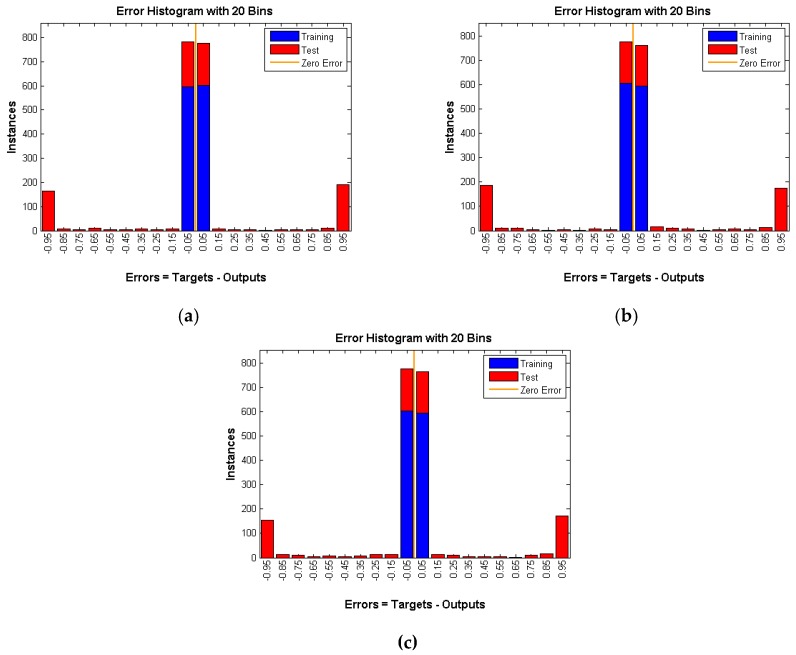
DAD with rate of (**a**) 0.05; (**b**) 0.1 and (**c**) 0.15.

**Figure 12:**
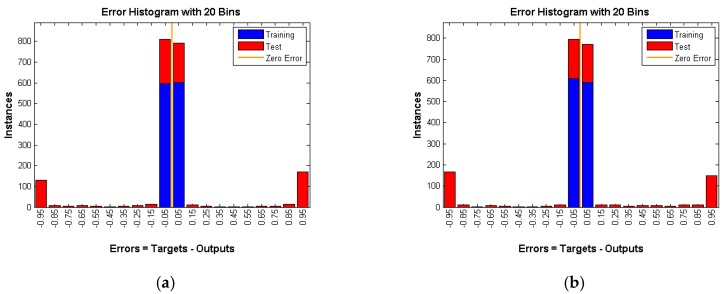

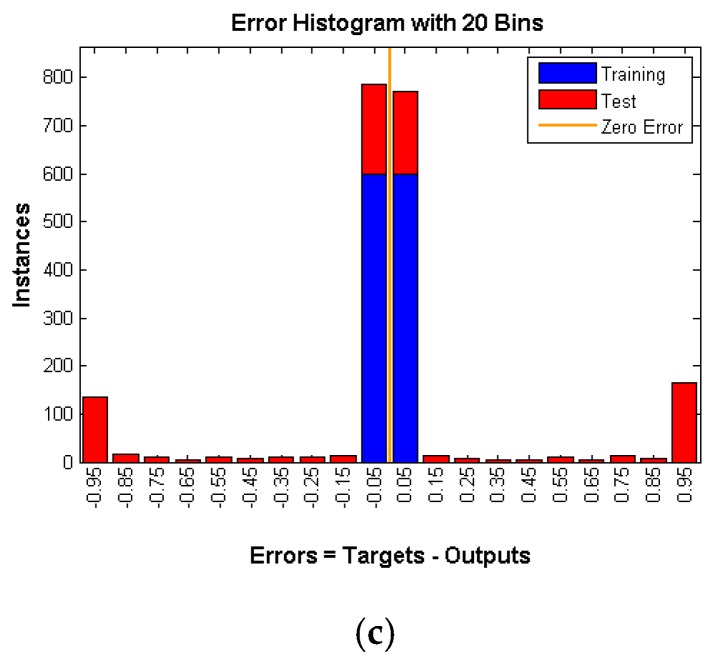
CAD with rate of (**a**) 0.05; (**b**) 0.1 and (**c**) 0.15.

As shown in Figure 10, Figure 11 and Figure 12 number of instances is the *y*-axis and errors are the *x*-axis. Errors are the difference between targets, which represent the expected outputs and outputs which represent the actual outputs. The error of the training data is almost 0, whereas the error of testing data is high and is around 50 percent (Table 1) in all three techniques under various rates. That means the neural network is confused about the existence of the real event (Panda) even after successful training of the network. This is a very strong indication that proposed techniques are very reliable and guarantee source anonymity.

### 7.3. Anonymity Equation

The ideal case when **d**(α,β) is equal to 0 indicates the system is perfectly secure. Therefore, the proposed techniques should provide **d**(α,β) very close to 0 to confirm the high anonymity of our techniques, since the neural network in the previous subsection created some probabilities of the false positive and false negative. These probabilities can be used to generate α and β, respectively.

Table 2 shows that **d**(α,β) is very close to 0 in all three techniques using different rates which satisfy a very small ε that is required by most of the applications in order to have a high level of anonymity.

All analysis models prove that the proposed techniques provide a high level of anonymity and the global adversary cannot recognize the existence of the real event. Subsequently, this leads to location and time privacy of the real event.

## 8. Conclusions

Given the lack of techniques that provide anonymity to the origin, this paper presents SLP techniques for improving the performance of the wireless sensor network while keeping a high level of anonymity against a global adversary which is capable of monitoring the entire traffic in the network. Previous approaches to SLP transmit real or fake packets at each and every interval, resulting in high overhead and power consumption (e.g., FACP). To improve upon this aspect, three different techniques, DUD, DAD and CAD, were developed to provide a low transmission rate while still maintaining anonymity. Many of the previous papers in the literature do not provide an approval of their technique’s performance. Therefore, all proposed techniques were tested under different analysis models to confirm that they provide a high level of anonymity. The simulations show that one of the techniques developed here, CAD, is the best in terms of average delay and delivery ratio while guaranteeing delivery of the event within a certain delay constraint. All three techniques have low overhead, i.e., use the same total number of packets. Results show that proposed techniques perform very well even under very low rates such as 0.05 in all analysis models, and provide low delay and high delivery while maintaining anonymity. 

## Figures and Tables

**Table 1:** Error Percentage for testing data.

Rate	DUD	DAD	CAD
0.05	50.4	49.4	56.5
0.1	48.1	48.6	53.0
0.15	52.3	51.3	53.4

**Table 2:** **d**(α,β) based on different α and β values.

Rate	DUD			DAD			CAD		
	α	β	d(α,β)	α	β	d(α,β)	α	β	d(α,β)
0.05	0.499	0.494	0.0001	0.508	0.504	0.0004	0.442	0.426	0.0507
0.1	0.521	0.516	0.0004	0.517	0.511	0.0023	0.472	0.468	0.0104
0.15	0.481	0.474	0.0058	0.491	0.486	0.0015	0.471	0.461	0.0134

## Abbreviations

DUD	Dummy Uniform Distribution
DAD	Dummy Adaptive Distribution
CAD	Controlled Dummy Adaptive Distribution
SLP	Source Location Privacy
FACP	Fortified Anonymous Communication Protocol
GFS	General Fake Source
